# High-Pressure Carbon Dioxide Use to Control Dried Apricot Pests, *Tribolium castaneum* and *Rhyzopertha dominica*, and Assessing the Qualitative Traits of Dried Pieces of Treated Apricot

**DOI:** 10.3390/foods10061190

**Published:** 2021-05-25

**Authors:** Reza Sadeghi, Fereshteh Heidari, Asgar Ebadollahi, Fatemeh Azarikia, Arsalan Jamshidnia, Franco Palla

**Affiliations:** 1 Department of Entomology and Plant Pathology, College of Aburaihan, University of Tehran, Tehran 3391653755, Iran; f.heidari4234@gmail.com (F.H.); jamshidnia@ut.ac.ir (A.J.); 2 Department of Plant Sciences, Moghan College of Agriculture and Natural Resources, University of Mohaghegh Ardabili, Ardabil 5619936514, Iran; 3 Department of Food Technology, College of Aburaihan, University of Tehran, Tehran 3391653755, Iran; azarikia@ut.ac.ir; 4 Department of Biological, Chemical and Pharmaceutical Sciences and Technologies, University of Palermo, 38-90123 Palermo, Italy

**Keywords:** apricot, CO_2_ gas, qualitative traits, warehouse pest

## Abstract

One of the new ways of warehouse pest control is the carbon dioxide treatment, which had no residues on the target products. In the present research, at first, CO_2_ gas was applied to control two important pest species infesting dried apricots. Dry apricots infested with adults of *Tribolium castaneum* (Herbst) or *Rhyzopertha dominica* (F.) were exposed to CO_2_ gas pressures correspond to 9.1, 16.7, 23.1, 28.6, and 33.4 mol% for 24 h. The results showed higher mortality rates with increasing the gas pressures in all the experiments. The minimum and maximum losses of the pests were determined at concentrations of 9.1 and 33.4 mol%, respectively. Evaluation of CO_2_ gas effects on the quality characteristics of dried apricots showed no impacts on the color, brittleness, hardness, sweetness, sourness, and general acceptance of products. CO_2_ gas treatments at the concentration of 33.4 mol% showed no significant influences on the chemical features of dried apricots, including pH, acidity, Brix, humidity percentage, reducing sugar, and total sugar. It was concluded that CO_2_ gas had the potential to control *T. castaneum* and *R. dominica* in warehouses of dried apricots, without any significant impacts on product qualities.

## 1. Introduction

Apricot, *Prunus armeniaca* L. (Rosales: Rosaceae), is considered as one of the most delicious fruits in regions with temperate climates. It contains saccharides, organic acids, minerals, vitamins, and polyphenols and has antioxidant properties [1]. Apricot is served fresh or as dried or frozen fruit, compote, extract, and jam [2]. According to the Food and Agriculture Organization (FAO) statistics, Turkey, Uzbekistan, and Iran ranked 1st to 3rd in the world in 2018 for producing 750,000, 493,842, and 342,479 tons of apricot, respectively [3]. Dried fruits are stored in warehouses under specific controlled conditions to maintain their quality and marketability, besides preventing pest infestation. Millions of tons of crops and dried fruits are annually lost because of the damage created by storage pests and the non-observance of scientific storage principles. Heavy quantitative, qualitative, and hygienic damages may be inflicted on storage products by insects [4]. To be supplied to the world markets, agricultural products must satisfy the necessary standards such as acceptable taste and color and particularly moisture content without any infestations of insects [5].

Conventional pest management practices provide such advantages as ease of operations and low costs; yet, overuse of synthetic chemicals involves some disadvantages such as prolonging slow operations, producing environmentally polluting and ozone-depleting wastes, imposing health risks on the operators, having adverse impacts on product quality, and so on [6,7,8]. For instance, methyl bromide has been successfully applied to control a variety of insects over the years, but due to the hazardous environmental impacts its use has been limited by international subsidiary organizations until 2015 [9]. Thus, it is necessary to replace synthetic insecticides with environmentally friendly agents concerning the problems related to chemical insecticides, besides the economic importance of pest control [10]. In previous publications, as an alternative to synthetic pesticides, interests have been focalized on plant essential oils [11,12].

Being a non-flammable and odorless gas with a body mass of 1.5 times the air mass, carbon dioxide (CO_2_) had no residues on target products while also being toxic to insects [13]. A wide range of pests, along with their different growth stages, can be controlled by CO_2_ gas during the storage of dried fruits [14]. An increase in the amount of CO_2_ would lead to enhancement of the respiratory rates of insects. Therefore, in warehouses, insects can be killed even at low CO_2_ concentrations, which results in the disruption of their breathing regulations when it enters their body tissues and fluid organs [15]. Additionally, respiratory pores of insects may be kept open at the concentration of 35% CO_2_, which can force extra water uptakes of their cellular tissues and impair the metabolism of organs by reducing amounts of triglycerides; these conditions would consequently lead to their mortalities [16]. Effectiveness of CO_2_ in the control of stored-product insect pests was assessed in the previous studies. For example, Cheng et al. [17] used a mixture of 2% oxygen and 18% carbon dioxide, which could stop all the growth stages of cowpea weevil, *Callosobruchus maculatus* F. In their study, Valizadegan et al. [18] were able to control four species of storage pests with a combination of phosphine and carbon dioxide, which provided the advantage of reduced phosphine consumption. Complete controls of the insects were achieved within 7, 10, and 11 weeks after 24 h-treatment. Carbon monoxide and dioxide efficacies were investigated by Dhouibi et al. [19] for controlling flour and carob moths (*Ephestia kuehniella* Zeller and *Ectomyelois ceratoniae* Zeller, respectively) infesting organic dates. Mortality of all tested insects was achieved by 5.5 and 2.8 kg/ m^3^ of CO_2_ fumigations after 20 and 48 h under laboratory and field conditions, respectively.

The color lab space was designed in a way to be very similar to what is perceived by human vision [20]. In the image processing technique, the parameters of L*, a*, and b* indicate brightness, redness, and yellowness of products, respectively, which can be changed in the treatments by pest control and preserving agents [21]. For example, Sadeghi et al. [22] reported increasing L*, a*, and b* variations for figs and raisins with enhancing microwave powers and timings in the sawtoothed grain beetle (*Oryzaephilus surinamensis* L.) management. Additionally, Inserra et al. [23] observed an increase in L* and b* and a reduction in a* parameters of apricots treated with sulfur before drying.

Although the red flour beetle (*Tribolium castaneum* Herbst) and the lesser grain borer (*Rhyzopertha dominica* (F.)) are among the cosmopolitan insect pests of stored flour and grains, they can damage dried material of animal and plant origin such as dried fruits [24,25]. This study aimed to (1) determine the effect of high-pressure CO_2_ on the mortality of two cosmopolitan stored-products insect pests *T. castaneum* and *R. dominica* and (2) investigate possible changes in sensory (organoleptic) and chemical properties of dried apricot affected after treatment by CO_2_.

## 2. Materials and Methods

### 2.1. Dried Fruit and Rearing Insect Pests

The stored product used in present investigation was dried apricot (*Prunus armeniaca* L., Shahroudi cultivar) purchased from the wholesale dried fruit market in Tehran City, Iran. *R. dominica* and *T. castaneum*, which had been previously reared for ten generations, were prepared in the Toxicology Laboratory of the Department of Entomology and Plant Pathology, College of Aburaihan, University of Tehran, Iran. The 1.5 kg of the two food compositions of broken cereal [26] and intact wheat grains [27] were utilized for breeding *T. castaneum* and *R. dominica*, respectively. Then, 200 insects of each pest (without sex separation) were released in 3-L plastic containers to be reared at the temperature of 27 ± 2 °C, relative humidity of 65% ± 5%, and light: dark of 10:14 h [28]. Afterward, a brush was used to remove 50 insects from the permanent foodstuff and place them on the rearing food in new containers. Adults were then separated and transferred to the main dish to avoid generational intermingling after a week of mating and spawning. After 27 days, the next adults of the insects appeared and 1–10 day-old adults were used in the experiments.

### 2.2. Fumigation of Carbon Dioxide

The pressures of CO_2_ gas, which was provided by 20-L capsules, were determined by the equipped pressure gauge during the preliminary tests. The experiment was laid out in a completely randomized design (CRD) with ten replications. Disposable paper cups, with the diameter and height of 7.5 and 8.7 cm, respectively, were used to hold 30 g of the dried apricot samples contaminated with twenty pests. After transferring the containers of each treatment to a cylindrical autoclave (Kavush Mega Medical Instrument Co., Tehran, Iran) with a 10-L stainless steel tank equipped with a pressure gauge, CO_2_ inlet and outlet valves, and closable steel lids to confine CO_2_, the lids were covered with lace and elastic bands. Air inside the autoclave was allowed to completely exit by opening the outlet valve after 20 s of fumigation. Then, the valve closed and fumigation was prolonged at the specified pressures from 0.1 to 0.5 bar correspond to 9.1, 16.7, 23.1, 28.6, and 33.4 mol%. Finally, 24 h after CO_2_ fumigation and aeration, the mortality of adult insects was counted. The control treatments underwent no pressure of CO_2_ gas.

### 2.3. Qualitative Properties

The samples of the dried apricot were studied for organoleptic properties, including color, aroma, sweetness, sourness, brittleness, hardness, and general acceptance, before and after CO_2_ treatments. Ten expert evaluators (5 males and 5 females) confirmed in the taste validity test according to the standards [29] were employed to assess the characteristics. The sensory properties of the apricot specimens were judged by the evaluators using the 7-point hedonic test. The numerical scales of 1–7 (higher qualitative levels with increasing numbers) were recorded in their given forms expressing the intensity of each qualitative attribute. The treated samples were then compared with the five control samples poured into the plastic containers. The evaluators were also provided with some disposable cups of mineral water to avoid interfering with the tastes of the samples between each detection step. These experiments were also arranged in a completely randomized design (CRD) in ten replications.

A digital camera (OLIMPUS VR-310, Tokyo, Japan) was employed to take photos of the pieces of apricots. An artificial lighting system was utilized to provide a uniform lighting condition for the specimens. It was done by using a white fluorescent lamp mounted under the ceiling of our special chamber, the walls of which were covered with a black cover. Photographing was performed at a distance of 30 cm from the specimens put against a white background [22]. The images were captured at 959 pixels × 1280 pixels and a resolution of 96 dpi in Jpeg format and RGB color space. Image 1.50 v software was applied to process the images captured by the digital camera.

### 2.4. Determination of Chemical Properties

First, the 3-g samples of the apricot pieces, in five replications and pretreated with CO_2_ gas at the concentration of 33.4 mol%, were stirred in 20 mL of distilled water using a magnetic stirrer at 80 °C. After 2 h, the liquid part was separated by filter paper [23]. To determine pH and Brix number, a pH meter and a refractometer were employed, respectively.

To specify the acidity, 10 mL of the prepared solution in five replications was titrated with NaOH (0.1 M) until obtaining a pale pink color (pH = 8.3). The results were reported in terms of dominant acid percentage: malic acid [23].

To delineate the moisture content, 3 g of the samples (in three replications) were placed in a glass plate heated by an oven to 80 °C for 18 h, and the percentage of moisture content was determined by dividing the difference of the sample weights before and after heating [30].

To determine the reducing sugar, 3 g of the sample (in three replications) were first mixed with 40 mL of distilled water at 80 °C and was filtered through filter paper after 2 h. Then, 5 mL of Fehling A and Fehling B together with a magnet and about 10 mL of distilled water were poured into a 100-mL Erlenmeyer flask. Afterward, the Erlen was placed on a magnetic stirrer with a heater and slowly titrated with the sugar solution until fading the blue color and appearing the brick color [31].

To specify total sugar, 25 mL of the filtered solution was first poured into a 100-mL volumetric flask and 5 mL of concentrated hydrochloric acid (37%) was added to it to be then heated in a boiling water bath (Bain Marie) at 65 °C for 10 min. After reaching ambient temperature, 2 drops of phenol phethalene reagent were added to the mixture, which was then neutralized with 45% concentrated sodium hydroxide (0.1) to give a pale pink color. Finally, it was volumized with distilled water and then titrated with the prepared sugar solution according to the method described for reducing sugar [31]. This experiment was also performed in three replications. All experiments about the evaluation of chemical properties of dried apricots were arranged in a completely randomized design (CRD).

### 2.5. Statistical Analysis

The Kolmogorov-Smirnov test was used to check the normality of the data. Analysis of variance (ANOVA) was used to determine the statistical effect of CO_2_ pressures on insect pests. Differences between means were assessed using the Tukey HSD test at *p* < 0.05. When any death was observed in the control groups, mortality percentages were corrected using Abbott’s formula [32]. Lethal concentration values (LC_50_ and LC_90_ with their 95% fiducial limits) and the regression line particulars were calculated using Probit analysis. All statistical analyses were performed with SPSS version 25 (IBM, Chicago, IL, USA). Evaluation of chemical properties (pH, acidity, Brix, total sugar, reducing sugar, and moisture) between treated and untreated dried apricot samples were compared using the *t*-test. A non-parametric Friedman’s test used for sensory assessment.

## 3. Results

The adults of *R. dominica* and *T. castaneum* were highly susceptible to the fumigation of CO_2_. According to the results of the analysis of variance, the mortality of *R. dominica* and *T. castaneum* adults was statistically affected by different concentrations of CO_2_. As can be seen in Table 1, the increasing CO_2_ concentration were associated with the enhanced mortality of the both insects. The CO_2_ concentration of 33.4 mol% caused 90 and 78% mortality of the adults of *R. dominica* and *T. castaneum*, respectively (Table 1).

Along with the high mortality of *R. dominica* at all tested concentrations of CO_2_, the calculated LC_50_ value (20.19 mol%) for this pest was also lower than that corresponding value for *T. castaneum* (27.02 mol%). However, based on the overlapping of their fiducial limits, the susceptibility of these insects to CO_2_ was not significantly different (Table 2). Additionally, according to the *R*^2^ values in Table 2, there was a direct and positive relationship between the tested CO_2_ concentrations and the observed mortality of both insects.

Table 3 shows the means of the sensory characteristics of the dried apricots and their comparison via the Friedman’s test. The results revealed that the different concentrations of CO_2_ gas had no significant impact on the participant’s preference for the color, hardness, brittleness, sweetness, and sourness of the dried apricots, while the participant preference for aroma and general acceptance were decreased at the concentrations of 28.6 and 33.4 mol% (Table 3).

**Figure 1:**
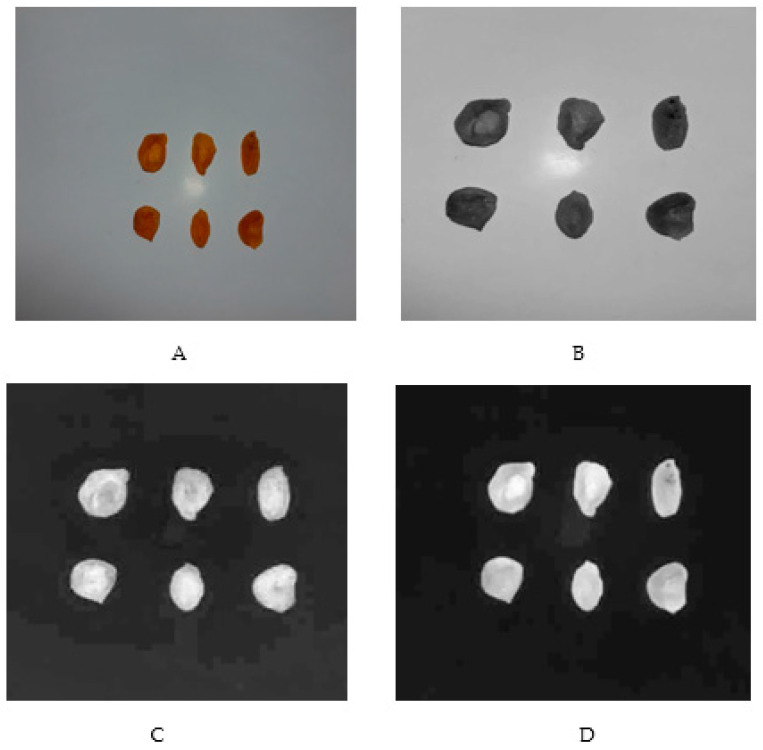
Image processing of the dried pieces of apricot: (**A**) RGB pictures taken with the digital camera; (**B**) Factor L* (brightness); (**C**) Factor a* (redness); (**D**) Factor b* (yellowness). In the images taken, the color parameters (L*, a*, and b*) were calculated for all pixels of the samples, and according to them, the mean values of each parameter were estimated (27.970, 23.119, and 36.397, respectively) through ImageJ 1.50 v software.

RGB pictures taken with the digital camera and colorimetric factors (L*, a*, and b*) of the dried apricot treated by CO_2_ were shown in Figure 1. In the images taken, the color parameters (L*, a*, and b*) were calculated for all pixels of the samples. Then the mean values of each parameter were estimated for each image. The values of the color parameters L*, a*, and b* were calculated as 27.970, 23.119, and 36.397, respectively.

**Figure 2:**
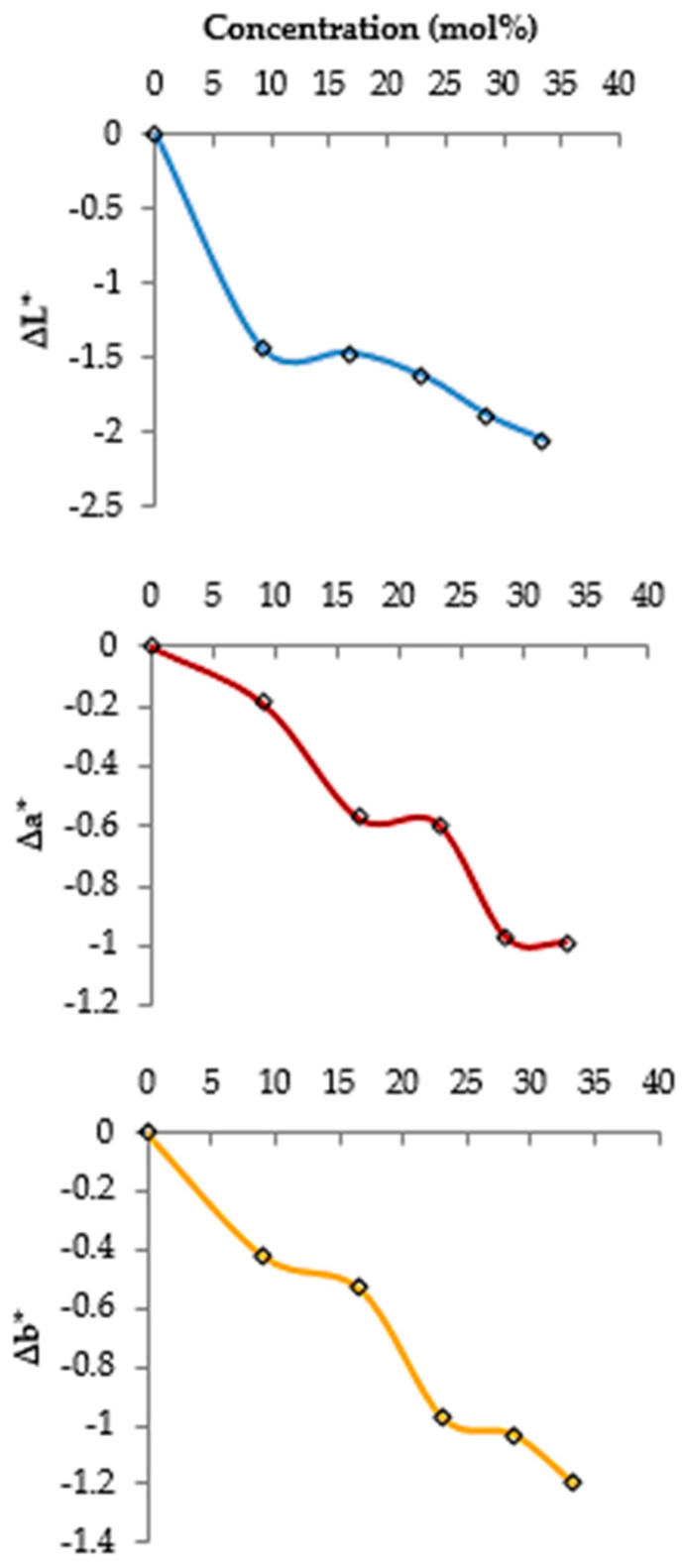
Effects of CO_2_ concentrations on the color parameters (L* (brightness), a* (redness), and b* (yellowness)) of the dried apricots.

Figure 2 displays the increased changes of the brightness (ΔL*), redness (Δa*), and yellowness (Δb*) of the treated samples with enhancing CO_2_ concentrations. Changes in the red color are almost constant at the concentrations between 16.7–23.1 and 28.6–33.4 mol%. Generally, alterations in the product color factors (L*, a*, and b*) induced by the CO_2_ treatments are trivial. This degree of color changes triggered by CO_2_ treatment is usually detectable with bare eyes.

Analysis of CO_2_ gas impact at the concentration of 33.4 mol% on the chemical properties of the dried apricots, including pH, moisture content, acidity, Brix number, reducing sugar, and total sugar, and comparison with those of the control group revealed no significant difference between them (Table 4). Therefore, CO_2_ gas at the concentration of 33.4 mol% would not affect the chemical properties of the control and treated dried pieces of apricot product.

## 4. Discussion

In current research, the mortality rates of two pest species were enhanced with increasing CO_2_ concentration. The highest mortality, 78.0% and 90.5% for the adults of *T. castaneum* and *R. dominica*, respectively, was at the concentration of 33.4 mol%. Kells et al. [33] noticed the increasing CO_2_ concentration from 25 to 50 ppm to enhance the larval mortality of Indian meal moth (*Plodia interpunctella* Hübner) from 77 to 94.5%. Riudavets et al. [34] reported that nine storage pests could be controlled by the CO_2_ pressures of 15 and 20 bar within the time periods of 15, 30, and 60 min. In their study, 5.5% and 100% of the mortality of *R. dominica* adults were observed at the pressures of 15 and 20 bar after 15 min-exposure time, respectively. Accordingly, considering about 90% mortality by 0.5 bar pressure (33.4 mol%) of CO_2_ in the present study, it will be possible to increase the mortality of *R. dominica* by increasing gas pressure (concentration) and exposure time. In research carried out by Sayeda and Hashem [35], the elevated mortality rate of Indian meal moth larvae from 38.9 to 77.8% was observed with increasing CO_2_ concentration from 40 to 80% during 24 h of fumigation. These researchers reported CO_2_ concentrations of 40% and 80% to lead to 35% and 71.7% larval mortality of almond moth (*Ephestia cautella* (Walker)), respectively. They experienced more vulnerabilities of the eggs and pupae of both species to the gas concentrations in comparison with larvae. At the concentration of 80%, 5 and 7 days required for full mortality of egg and pupal and larvae, respectively [35]. In the study of Wong-Corral et al. [36], increasing CO_2_ concentration led to the enhanced mortality of Mexican bean weevil, *Zabrotes subfasciatus* (Boheman). After 2 days of treatment, gas concentration of 90% resulted in complete mortality, while the concentrations of 70 and 50% were required for complete mortality after 3 and 4 days of fumigation, respectively. However, they found that the adults of cowpea weevil and bean bruchid (*Acanthoscelides obtectus* (Say)) were more susceptible to the gas concentrations than Mexican bean weevil adults. In the research of Sadeghi et al. [37], the ozone concentration of 2 ppm and time period of 15 min resulted in the lowest mortality of the adults of the sawtoothed grain beetle (*Oryzaephilus surinamensis* L.) and larvae of flour moth *E. kuehniella*, while the concentration of 5 ppm and time of 90 min led to complete mortalities. These findings, about the susceptibility of stored-products insect pests to CO_2_ gas, supports the results of current investigation. This is also congruent with our results, which showed increased mortality of the storage insects by elevating CO_2_ gas concentrations.

In the present study, no significant impacts on the color, aroma, sweetness, sourness, brittleness, hardness, and general acceptance of the dried apricot were caused by enhancing the gas concentration of CO_2_. Similarly, Sadeghi et al. [37] reported no significant influences of increasing ozone concentration and times of fumigation on the color, sweetness, sourness, brittleness, hardness, and general acceptance of dried figs and raisins, but they witnessed considerable effects on the aroma of the two products. Of course, they concluded that due to the volatility of ozone gas, their unpleasant odors could disappear over time. Accordingly, documented effects of CO_2_ concentrations on the aroma of dried apricots can be removed over time. In the test of color changes of the product surface, the increases in CO_2_ gas pressures were observed to have trivial effects on the surface colors of the dried apricot samples, which was not detectable with bare eye. In this regard, Sadeghi et al. [37,38] reported that increasing ozone concentrations (2, 3, and 5 ppm) and exposure times (15, 30, 45, 60, and 90 min) had no significant effects on the colorimetric factors of their raisins and figs.

Furthermore, CO_2_ gas at the concentration of 33.4 mol% had no impacts on the chemical properties (pH, Brix value, acidity, reducing sugar, total sugar, and moisture content) of the dried apricots. These results were consistent with the findings of Inserra et al. [23], who reported insignificant impacts of sulfur on the moisture content, Brix value, and total sugar of dried apricot. The moisture percentage, pH, acidity, Brix value, reducing sugar, and total sugar contents of the dried apricots (Alkia cultivar) treated with sulfur were measured as be 22.5%, 4.2%, 1.8%, 68.1%, 64.4%, and 87.9%, respectively. These results, regarding no significant differences in chemical properties of treated and untreated dried apricot, are in agreement with those obtained in our study. The reason for the discrepancy of the measured Brix values in the study of Inserra et al. [23] and present study (68.1% and 9.82%, respectively) is related to the differences in the measurement methods, i.e., amounts of water added to the samples. In addition, the varied moisture contents can be attributed to the differences in the durations of the drying methods and their varieties [23].

## 5. Conclusions

According to the present results, fumigation of CO_2_ caused significant toxicity on the adults of *R. dominica* and *T. castaneum* so that 20.19 and 27.02 mol% of the gas were adequate to kill 50% of pests within 24 h, respectively. The use of CO_2_ gas, despite the toxicity to insect pests, had no significant side-effects on the sensory and chemical properties of the treated apricot specimens. Consequently, the prospective insecticidal activity of CO_2_ gas offers an efficient tool to control the cosmopolitan insect pests *R. dominica* and *T. castaneum*, with no detrimental side-effects on stored-apricots. The main advantage of using carbon dioxide in preserving dried fruits such as apricots from pests is no necessity of complex and expensive devices. In other words, by using the cylinders containing this gas and adjusting the desired pressure, along with an impermeable covering of the warehouse, the control procedure can be carried out satisfactorily [39].

## Figures and Tables

**Table 1:** Means ± standard errors of the mortality rates of *T. castaneum* and *R. dominica* at different CO_2_ pressures.

Concentration (mol%)	Mortality Percentage + SE	
	*T. castaneum*	*R. dominica*
Control = 0	1.00 ± 0.667 ^a^	3.50 ± 1.302 ^a^
9.1	5.50 ± 1.167 ^a^	23.00 ± 1.106 ^b^
16.7	9.50 ± 1.572 ^a^	44.00 ± 2.667 ^c^
23.1	27.50 ± 3.184 ^b^	57.50 ± 2.713 ^d^
28.6	57.00 ± 1.700 ^c^	80.00 ± 2.108 ^e^
33.4	78.00 ± 3.000 ^d^	90.50 ± 3.114 ^f^
ANOVA:	F = 223.54 df = 5, 54 *p* = 0 < 0.001 *	F = 210.23 df = 5, 54 *p* = 0 < 0.001 *

Values with different letters within each column are statistically different according to the Tukey’s test at *p* < 0.05. Asterisks indicate significant effects of CO_2_ concentrations on mortality of insect pests, according to the results of the analysis of variance (ANOVA). SE is standard error.

**Table 2:** Probit analysis of the toxicity of CO_2_ on the adult-insects of *R. dominica* and *T. castaneum*.

Insect	LC_50_ with 95% Fiducial Limits (mol%)	LC_90_ with 95% Fiducial Limits (mol%)	Intercept ± SE	Slope ± SE	*χ*^2^ (df = 3)	Sig.	*R*^2^ Value
*R. dominica*	20.19 (18.37–22.19)	54.45 (38.41–131.89)	−3.88 ± 0.43	2.97 ± 0.32	5.58	0.13	0.95
*T. castaneum*	27.02 (18.42–104.51)	48.26 (32.83–5320.19)	−7.21 ± 0.67	5.03 ± 0.49	26.19	0 < 0.001	0.87

Number of tested adults for each species was 500. Sig. is significant.

**Table 3:** Mean panelists ranking scores of the sensory properties of the dried apricot treated with different concentration of CO_2_.

Concentration (mol%)	Aroma	Color	Hardness	Brittleness	Sweetness	Sourness	General Acceptance
Control = 0	3.80 ± 0.20 ^b^	5.10 ± 0.23 ^a^	2.10 ± 0.18 ^a^	2.40 ± 0.26 ^a^	4.30 ± 0.15 ^a^	3.30 ± 0.26 ^a^	4.80 ± 0.29 ^c^
9.1	3.70 ± 0.26 ^b^	5.10 ± 0.23 ^a^	2.00 ± 0.21 ^a^	2.10 ± 0.31 ^a^	4.40 ± 0.26 ^a^	3.20 ± 1.35 ^a^	4.90 ± 0.23 ^c^
16.7	3.60 ± 0.22 ^b^	4.90 ± 0.23 ^a^	2.00 ± 0.21 ^a^	2.00 ± 1.33 ^a^	4.10 ± 1.37 ^a^	3.60 ± 1.47 ^a^	4.30 ± 0.26 ^bc^
23.1	3.70 ± 0.21 ^b^	4.90 ± 0.18 ^a^	2.10 ± 0.21 ^a^	1.90 ± 0.27 ^a^	3.50 ± 1.45 ^a^	3.60 ± 1.42 ^a^	4.20 ± 0.29 ^bc^
28.6	2.60 ± 0.26 ^a^	4.70 ± 1.36 ^a^	2.20 ± 0.21 ^a^	1.90 ± 0.27 ^a^	4.633 ± 0.56 ^a^	3.50 ± 0.42 ^a^	3.40 ± 0.34 ^ab^
33.4	2.00 ± 0.25 ^a^	4.90 ± 0.27 ^a^	2.20 ± 0.13 ^a^	2.20 ± 0.29 ^a^	4.30 ± 0.36 ^a^	3.60 ± 0.34 ^a^	2.40 ± 0.26 ^a^
Friedman’s test:	*χ*^2^ = 26.83 df = 5 *p* = 0.0000006	*χ*^2^ = 2.22 df = 5 *p* = 0.817	*χ*^2^ = 1.63 df = 5 *p* = 0.898	*χ*^2^ = 1.87 df = 5 *p* = 0.867	*χ*^2^ = 4.83 df = 5 *p* = 0.963	*χ*^2^ = 1.67 df = 5 *p* = 0.894	*χ*^2^ = 28.67 df = 5 *p* = 0.000002

Means (± standard error) with different letters within each column are statistically different according to the Friedman’s test.

**Table 4:** Means ± standard errors of the chemical properties of the control and treated dried pieces of apricot after CO_2_ gas treatment at the concentration of 33.4 mol%.

Chemical Properties	Control	Treatment	Comparison of Means (df = 4)	
			t	Sig.*
pH	4.17 ± 0.17	4.30 ± 0.08	−2.42	0.73
Acidity	1.13 ± 0.29	1.19 ± 0.27	−0.92	0.41
Brix	9.96 ± 0.44	9.82 ± 0.46	0.39	0.72
Total sugar	78.29 ± 3.07	78.79 ± 3.92	−0.14	0.9
Reducing sugar	56.86 ± 3.72	57.70 ± 4.67	−0.20	0.86
Moisture	10.30 ± 0.30	10.43 ± 1.70	−0.14	0.9

* Since the significant value is more than 0.05, tested means have no significant difference in each control and treatment row, according to the *t* test at *p* < 0.05. df and sig. are degree of freedom and significant, respectively.

## Data Availability

The data that support the findings of this study are available upon request from the authors.
